# Docosahexaenoic acid (DHA) alleviates hepatic lipid deposition in dairy cows during the transition period: an integrated in vitro and in vivo study

**DOI:** 10.1186/s40104-025-01308-4

**Published:** 2025-12-05

**Authors:** Xinyue Zhang, Xiaojing Liu, Siyuan Liu, Weixuan Tang, Shaoxiong Ji, Hongjin Ji, Ya Jing Wang, Zhijun Cao, Hongjian Yang, Wei Wang, Shengli Li

**Affiliations:** State Key Laboratory of Animal Nutrition and Feeding, Department of Animal Nutrition and Feed Science, College of Animal Science and Technology, China Agricultural University, Beijing, 100193 China

**Keywords:** Dairy cow, Docosahexaenoic acid, Fatty liver, Lipid metabolism, Mitochondria

## Abstract

**Background:**

Fatty liver syndrome is a prevalent metabolic disorder in transition dairy cows, characterized by excessive hepatic lipid accumulation that impairs liver function and leads to systemic metabolic disturbances. Docosahexaenoic acid (DHA), a prominent n-3 polyunsaturated fatty acid (PUFA), not only exhibits anti-inflammatory and anti-oxidative properties, but also holds potential in ameliorating lipid metabolism. This study integrated in vitro bovine primary hepatocyte models and in vivo dairy cow trials to investigate the regulatory effects of DHA on hepatic lipid deposition.

**Results:**

In vitro, 40 μmol/L DHA significantly reduced triglyceride (TAG) accumulation in steatotic hepatocytes by downregulating genes involved in fatty acid transport (*FABP-1*, *CD36*) and lipogenesis (*DGAT2*, *FAS*, *SREBP-1C*), while upregulating markers of lipolysis (*CGI-58*, *ATGL*) and fatty acid oxidation (*ACADL*, *CPT1A*, *CPT2*). Transmission electron microscopy (TEM) confirmed DHA-mediated restoration of mitochondrial ultrastructure and enhanced lipid droplet (LD)-mitochondria interactions. In vivo, dietary rumen-protected DHA (180 g/d) supplementation reduced hepatic lipid deposition, improved liver function (evidenced by decreased total bilirubin and alanine aminotransferase), reduced oxidative stress and inflammation (suppressed malondialdehyde, glutathione peroxidase, and lipopolysaccharide), coincided with relieving insulin resistance (reduced insulin and glucose, as well increased adiponectin) in dairy cows with fatty liver. These improvements may be attributed to increased expression of TOMM20 and MtCo-1, promoting mitochondrial biogenesis and β-oxidation, along with an elevated plasma n-3/n-6 ratio.

**Conclusions:**

Collectively, these findings suggest that DHA supplementation represents a promising nutritional strategy for preventing spontaneous fatty liver in transition dairy cows by enhancing hepatic lipid clearance and restoring metabolic homeostasis.

**Supplementary Information:**

The online version contains supplementary material available at 10.1186/s40104-025-01308-4.

## Introduction

The transition period is a critical stage in the lifecycle of dairy cows. During the final three weeks of gestation, nutritional demands for fetal growth, mammary gland development, and colostrum synthesis increase sharply, coinciding with a 10%–30% voluntary decrease in dry matter intake (DMI) [1, 2]. Following calving, a rapid surge in milk yield exacerbates the negative energy balance (NEB), leading to the mobilization of body reserves [3]. Consequently, free fatty acids (FFAs) increase in circulation, around one-third of which are absorbed by liver and re-esterified to triacylglycerol (TAG), contributing to hepatic lipid droplet (LD) accumulation and triggering the onset of fatty liver syndrome [4–6]. Data suggested that more than half of the cows may experience moderate to severe hepatic lipidosis [7], indicating a high prevalence of metabolic-associated fatty liver disease (MAFLD) in dairy production.

As the central organ of energy metabolism, the liver is particularly vulnerable to lipid overload. Excessive hepatic lipid accumulation disrupts liver function, and is often accompanied by insulin resistance, inflammation, and oxidative stress, all of which adversely affect milk yield, fertility, and longevity, ultimately resulting in substantial economic losses in the dairy industry [5, 8]. To date, dietary modulation has been regarded as a more sustainable strategy than pharmaceutical intervention for preventing hepatic lipid deposition [9, 10]. Recent study has uncovered the potential of dietary essential amino acids, especially leucine and isoleucine in alleviating hepatic steatosis [11]. Likewise, Markova et al. [12] reported that high-protein diets reduced liver lipidosis by 36%–48% in patients with MAFLD within 6 weeks. Among nutritional interventions, it is worth mentioning that n-3 polyunsaturated fatty acids (n-3 PUFAs), an essential FA that cannot be synthesized de novo by mammals [13], have attracted considerable attention on metabolic diseases based on their anti-inflammatory and antioxidant properties [14, 15]. More specially, as the predominant of n-3 PUFAs, docosahexaenoic acid (DHA) has shown promising beneficial effects in reducing obesity and metabolic syndrome [16]. In rodent model, DHA intervention significantly reduced liver TAG content and LD accumulation [17–19]. Another trial in children with MAFLD supported the clearance of liver lipid deposition at dose of 250 mg/d and 500 mg/d DHA [20]. While DHA has demonstrated lipid-lowering effects in monogastric animals, its application in ruminants remains underexplored. Existing studies have predominantly focused on DHA's efficacy in developing functional milk or dairy byproducts in lactating dairy cows [21, 22], whereas ignoring its potential in preventing fatty liver in transition dairy cows, particularly the mechanisms by which subcellular dynamics modulate lipid homeostasis. This critical knowledge gap has hindered the precise application of DHA in nutritional interventions for ruminants.

Above all, this study aims to investigate the effects of DHA on hepatic lipid metabolism by integrating in vitro primary hepatocyte model with in vivo feeding trial in transition dairy cows. The hypothesis of this study was that dietary DHA supplementation would effectively prevent fatty liver development by restoring mitochondrial function, modulating lipid metabolism pathways, and altering fatty acid profiles, thereby mitigating hepatic inflammation and oxidative stress in postpartum dairy cows.

## Methods

### Primary hepatocyte isolation, culture and treatment

Primary hepatocytes were isolated from 35–45 kg neonatal Holstein calves acquired from Shounong Animal Husbandry Technology Co., Ltd. (Beijing, China), confirmed to be clinically healthy through comprehensive veterinary examination and subjected to a 12-h overnight fasting. Calves were euthanized under terminal anesthesia using xylazine hydrochloride (Shengxin, China; 0.3 mL, intramuscular), followed by systemic anticoagulation via jugular vein injection of heparin sodium (Solarbio, China, 1,500 IU/kg). Then, the hepatic caudate lobe was resected aseptically using a standardized hepatectomy protocol, and immediately transferred to a biosafety cabinet (Airtech, Suzhou Antai Airtech Co., Ltd., Suzhou, China) for primary hepatocyte isolation under sterile condition.

An optimized two-step collagenase perfusion protocol was performed [23, 24]. In brief, all perfusion solution were pre-warmed to 37 °C prior to use, perfusion solution A (140 mmol/L NaCl, 6.7 mmol/L KCl, 10 mmol/L HEPES, 2.5 mmol/L Glucose, and 0.5 mmol/L EDTA; pH = 7.4) was used to remove blood and initiate portal vein cannulation (10–15 min, 50 mL/min), followed by perfusion solution B (identical composition except with 5 mmol/L CaCl_2_ and no EDTA) for calcium-mediated cell adhesion (5 min, 50 mL/min). Finally, digestion and dissociation were performed with perfusion solution C (perfusion solution B + 0.2 g/L collagenase IV) at 20 mL/min for 15 min until tissue friability and perfusate turbidity, immediately terminated with chilled (4 °C) fetal bovine serum (FBS, Every Green, China).

After removing vessels, capsule, and connective tissues, the remaining liver was shredded and resuspended in pre-cooled (4 °C) RPMI-1640 medium (Gibco, USA), then filtering through a sequential mesh series: 50- (once), 100- (twice) and 200-mesh (twice), separately. Next, the obtained hepatocytes were cleaned thrice (500 × *g*, 3 min) with pre-cooled RPMI-1640 medium, red blood cells were removed during the second wash using a lysing buffer (Solarbio, China). Intact hepatocytes were isolated by single-density gradient centrifugation in alkaline medium and purified by additional thrice centrifugation with pre-cooled RPMI-1640 medium. Finally, cell viability was assessed using trypan blue exclusion assay (Thermo Fisher Scientific, USA), followed by seeding in 6-well plates (Labselect, China) using pre-warmed adherent medium (RPMI-1640 medium supplemented with 10% premium FBS, 1% penicillin–streptomycin, 10^–6^ mol/L insulin, 10^–6^ mol/L dexamethasone and 10 μg/mL vitamin C) at 37 °C and 5% CO_2_. After 4 h, the adhesion medium was replaced with growth medium (RPMI-1640 supplemented with 10% premium FBS and 1% penicillin–streptomycin).

After incubation in growth medium for 24 h, primary bovine hepatocytes were starved in RPMI-1640 medium for 6 h before treatment. In the preliminary trial, four concentration gradients of palmitic acid (PA) and oleic acid (OA) mixture (100, 200, 400, 800 μmol/L) at a ratio of 1:2 for 12 h were estimated for fatty liver model in RPMI-1640 medium containing 2% BSA (Sigma-Aldrich, USA), then four concentration gradients of DHA (20, 40, 80, 160 μmol/L) for 12 h were evaluated for therapy in growth medium. The mixed fatty acids were dissolved in NaOH, while DHA was dissolved in DMSO prior to supplementation. Based on intracellular TAG content (three repetitions) and cell viability (ten repetitions), the optimal concentrations of mixed fatty acids for fatty liver model establishment and DHA for therapy were selected for subsequent experiments. In summary, the formal trial included three groups: the untreated control group (C), the mixed fatty acids-induced fatty liver group (T), and the DHA intervention group based on fatty liver model (TD).

### Animal experiment and dietary treatments

This experiment was carried out according to national care regulations and were received the approval of the ethic committee from China Agricultural University (Beijing, China; Permission ID: AW61705202-1-03). Holstein cows used in current study were selected from Sunlon Livestock Development Co., Ltd. (Xingtai, China). After screening of 248 close-up dairy cows, a total of 60 individuals were enrolled at 21 days prepartum based on predicted calving date. Animals were selected by body condition score (BCS) and hepatic ultrasonography (Beijing Eastern Bell Technology Group, Beijing, China) into predicted healthy control (C group: BCS = 3.0–3.25, hepatic health grade Ⅰ–Ⅱ; *n* = 20) and predicted fatty liver group (BCS ≥ 3.5, hepatic health grade Ⅲ–Ⅳ; *n* = 40) at 21 days prepartum. The guideline of hepatic health grade was described in Fig. 3. The control group (C) received no treatment, but half of the predicted fatty liver cows were further assigned to TD group (*n* = 20) and supplemented with rumen-protected Schizochytrium powder (DHA: 16%; Xiamen Huison Biotech Co., Ltd., Xiamen, China). The supplementation started at 30 g/d per cow from 21 days prepartum and gradually acclimated to 180 g/d per cow by calving, then maintained at this level until 21 days postpartum. No treatment was given to the remaining predicted fatty liver cows (T group, *n* = 20). The rumen-protected Schizochytrium powder was processed using fermentation-based encapsulation technology to ensure rumen bypass. It was dissolved in water and orally supplied to the TD group cows. The content of medium and long-chain fatty acids in the additive was list in Table 1.

**Table 1:** The medium and long-chain fatty acid content of rumen-protected Schizochytrium powder

Name	Abbreviation	Content, ng/g
Caprylic acid	C8:0	5,456.7
Decanoic acid	C10:0	973.1
Hendecanoic acid	C11:0	46.3
Dodecanoic acid	C12:0	2,183.9
Tridecanoic acid	C13:0	118.9
Tetradecanoic acid	C14:0	4,522.0
Myristoleic acid	C14:1	1,006.2
Myristelaidic acid	C14:1 T	82.4
Pentadecanoic acid	C15:0	2,653.0
*cis*-10-Pentadecenoic acid	C15:1	2,509.7
*trans*-10-Pentadecenoic acid	C15:1 T	190.4
Hexadecanoic acid	C16:0	145,174.3
Palmitoleic acid	C16:1	41,879.7
Palmitelaidic acid	C16:1 T	1,537.3
Heptadecanoic acid	C17:0	1,360.8
*cis*-10-Heptadecenoic acid	C17:1	1,678.2
*trans*-10-Heptadecenoic acid	C17:1 T	157.6
Octadecanoic acid	C18:0	57,221.0
Petroselaidic acid	C18:1(n-12)T	74,798.0
Elaidic acid	C18:1(n-9)T	72,730.7
Linoleic acid	C18:2(n-6)	78,335.6
Linoelaidic acid	C18:2(n-6)T	544.3
*trans*-7-Nonadecenoic acid	C19:1(n-12)T	2,932.1
Arachidic acid	C20:0	4,363.0
gamma-Linolenic acid	C18:3(n-6)	323.9
*cis*-11-Eicosenoic acid	C20:1	48.7
*trans*-11-Eicosenoic acid	C20:1 T	1,798.8
alpha-Linolenic acid	C18:3(n-3)	1,557.7
Heneicosanoic acid	C21:0	437.9
*cis*-11,14-Eicosadienoic acid	C20:2	485.1
Docosanoic acid	C22:0	2,053.8
homo-gamma-Linolenic acid	C20:3(n-6)	148.1
Erucic acid	C22:1	25.6
Brassidic acid	C22:1 T	186.6
*cis*-11,14,17-Eicosatrienoic acid	C20:3(n-3)	159.3
Arachidonic acid	C20:4	552.2
Tricosanoic acid	C23:0	1,150.6
*cis*-13,16-Docosadienoic acid	C22:2	90.3
*cis*-5,8,11,14,17-Eicosapentaenoic acid	C20:5	3,870.4
*cis*-7,10,13,16-Docosic acidtraenoic acid	C22:4	98.7
*cis*-7,10,13,16,19-Docosapentaenoic acid	C22:5(n-3)	26,092.4
*cis*-4,7,10,13,16-Docosapentaenoic acid	C22:5(n-6)	221.3
Tetracosanoic acid	C24:0	4,495.3
Nervonic acid	C24:1	92.4
*cis*-4,7,10,13,16,19-Docosahexaenoic acid	C22:6	104,691.0

Blood samples were collected at days −21, 3, and 21 relative to calving. Liver biopsies were conducted once between 7 and 14 days postpartum. Milk yield was recorded daily and collected at postpartum day 21 for fatty acid analysis. After excluding animals with preterm or post-term delivery, diseases or injuries, 12 dairy cows per group were included in the final analysis. All animals were offered total mixed ration (TMR) twice daily ad libitum (0700 and 1500 h) with 3%–5% orts, and the diet ingredients and nutrient contents are detailed in Tables 2 and 3.

**Table 2:** The ingredients and nutrient contents of diet for prepartum dairy cows

**Items**	Content
Ingredients, %	
DDGS^a^	6.3
Soybean hulls	3.7
Wheat straw	4.8
Oat hay	7.0
Whole corn silage	62.2
Corn meal	3.9
Wheat bran	2.9
Soybean meal	1.8
Canola meal	5.2
Premix^b^	2.2
Nutrient contents, %DM	
Dry matter	53.7
Crude protein	14.9
Neutral detergent fiber	41.0
Acid detergent fiber	24.9
NE_L_, Mcal/kg^c^	1.5

^a^*DDGS* Distillers dried grains with solubles

^b^Additives per kilogram of premix: 174 KIU vitamin A, 29 KIU vitamin D, 532 IU vitamin E, 223.2 g Ca, 108.7 g P, 352.5 g K, 71.6 g Cl, 1.6 g Zn

^c^ NE_L_ represents the net energy for lactation and is calculated based on NRC (2001) [25]

**Table 3:** The ingredients and nutrient contents of diet for postpartum dairy cows

**Items**	Content
Ingredients, %	
Corn meal	8.1
Soybean meal	9.7
Canola meal	1.6
Flaked corn	4.9
DDGS^a^	1.6
Alfalfa hay	7.5
Whole cottonseed	1.6
Rumen-protected fatty acid calcium	0.6
Fat power	0.9
Beet pulp pellets	1.0
Sugarcane molasses	1.6
Yeast culture	1.6
Wet brewers' grains	3.2
Whole corn silage	50.1
Oat hay	2.6
Premix^b^	3.4
Nutrient content, %DM	
Dry matter	58.6
Crude protein	17.6
Neutral detergent fiber	25.4
Acid detergent fiber	15.0
NE_L_, Mcal/kg^c^	1.7

^a^*DDGS* Distillers dried grains with solubles

^b^Additives per kilogram of premix: 174 KIU vitamin A, 29 KIU vitamin D, 532 IU vitamin E, 223.2 g Ca, 108.7 g P, 352.5 g K, 71.6 g Cl, 1.6 g Zn

^c^NE_L_ represents the net energy for lactation and is calculated based NRC (2001) [25]

### Oil Red O staining

Fresh hepatocytes and liver tissues were fixed in 4% paraformaldehyde (Servicebio, China) for 24 h, embedded in OCT compound, sectioned, and strained with Oil Red O for lipid visualization, followed by hematoxylin counterstaining.

### Blood and cell supernatant analysis

Blood samples were obtained from the tail vein prior to morning feeding. Serum was maintained at room temperature and plasma on ice. After centrifuging at 2,000 × *g* for 10 min at 4 °C, supernatants were harvested and stored at −80 °C for further analysis. Assays for biochemical and inflammatory characteristics in blood and cell culture supernatants were performed by Beijing Laibotairui Technology Co., Ltd. (Beijing, China) using commercially available kits. The detailed commercial assay information were represented in Table S1.

### RNA isolation and quantitative PCR measurement

Total RNA of hepatocytes and liver tissues was extracted using TRIzol reagent (Invitrogen, USA). RNA purity and concentration were measured using a microplate reader (EPOCH2, BioTek, USA), and samples with A_260_/A_280_ ratios of 1.8–2.1 were used for further detection. cDNAs were synthesized using a BeyoRT™ Ⅱ cDHA reagent kit (Beyotime, China), and quantitative PCR was performed with BeyoRT™ SYBR Green qPCR Mix (2X, Low ROX, Beyotime, China) on a QuantStudio 6 Flex Real-Time System (Thermo Fisher Scientific, USA), with the following thermal profile: 95 °C for 2 min, followed by 40 cycles of 95 °C for 15 s and 60 °C for 20 s, then 95 °C for 15 s, 60 °C for 15 s and 95 °C for 15 s. The expression of target genes was normalized by internal reference GAPDH and calculated using the 2^−ΔΔCt^ method. Primer sequences of targeted genes were designed using Primer5 software (V 5.5.0) and synthesized in Sangon Biotech (Shanghai, China), then verifying specificity via Design and Analysis software (V 2.8.0). Table S2 shows the primer sequences used in this experiment.

### Western blot assay

Hepatocytes and liver tissues were lysed using RIPA buffer (Solarbio, China) supplemented with protease and phosphatase inhibitors (Beyotime, China). After centrifugation at 12,000 × *g* for 10 min at 4 °C, the supernatant was carefully aspirated and standardized using the BCA assay kit (Beyotime, China). Next, protein was denatured in SDS-PAGE (Beyotime, China) at 100 °C for 10 min, followed by electrophoresis (80 V, 15 min and 120 V, 1 h) and wet-turn transfer (250 A, 150 min) to polyvinylidene difluoride (PVDF, 0.45 μm, Millipore, USA) membranes, which were blocked with 5% skimmed milk (Biosharp, China) for 2 h at room temperature, incubated overnight with primary antibodies at 4 °C, then secondary antibody for 50 min at room temperature, and visualized using enhanced chemiluminescence (ECL) substrate (Meilunbio, China). Images were captured with a ChemiDoc XRS + system (Bio-Rad, USA) and analyzed by ImageJ software (V 1.53). Antibody details are provided in Table S3.

### Transmission electron microscopy (TEM) observation

Hepatocytes were fixed in pre-chilled TEM fixative (Servicebio, China) at 4 °C for 24 h. Afterwards, samples were washed thrice with 0.1 mol/L phosphate buffer (PB, pH = 7.4), post-fixed with 1% OsO_4_ (Ted Pella Inc, USA) in 0.1 mol/L PB for 2 h at room temperature in dark, rinsed thrice in PB, sequentially dehydrated in graded ethanol (30%, 50%, 70%, 80%, 95%, and 100% ethanol) and acetone (20 min per step), infiltrated with EMBed-812 (SPI, USA) resin mixtures for overnight at 37 °C, then polymerized at 65 °C for 48 h. Finally, ultrathin Sects. (60–80 nm) were cut using a Leica UC7 ultramicrotome, stained with 2% uranyl acetate (8 min, dark) and 2.6% lead citrate (8 min, CO_2_-free), and grids were air-dried overnight prior to TEM (Hitachi, HT7800/HT7700) imaging and analysis.

### Fatty acid profile analysis

The quantitative fatty acids were measured using an ultra-high performance liquid chromatography coupled to tandem mass spectrometry (UHPLC–MS/MS) system (ExionLC™ AD UHPLC-QTRAP^®^ 6500+, AB SCIEX Corp., Boston, MA, USA) in Novogene Co., Ltd. (Beijing, China). The samples were homogenized with isopropanol/acetonitrile (1:1) and ultrasound treatment for 10 min. After 60 min placed at −20 °C, centrifuged at 13,000 × *g* for 15 min. The supernatant (50 μL) was added to derivatization reagent (150 μL) and derivatized at 40 °C for 40 min. The sample was diluted by isopropanol/acetonitrile (1:1). Then supernatant (95 μL) was homogenized with 5 μL mixed internal standard solution and injected into the LC–MS/MS system for analysis. Separation was performed on a Waters ACQUITY UPLC BEH C18 column (2.1 mm × 100 mm, 1.7 μm) which was maintained at 40 °C. The mobile phase, consisting of 0.1% formic acid in acetonitrile/water (1:1) (solvent A) and isopropanol/acetonitrile (1:1) (solvent B), was delivered at a flow rate of 0.30 mL/min. The solvent gradient was set as follows: initial 45% B, 1 min; 45%–70% B, 4.5 min; 70%–75% B, 9 min; 75%–80% B, 12.5 min; 80%–100% B, 14 min; 100%–45% B, 15.1 min; 45% B, 17 min. The mass spectrometer was operated in negative multiple reaction mode. Parameters were as follows: ionspray voltage (−4,500 V), curtain gas (35 psi), ion source temp (550 °C), ion source gas of 1 and 2 (60 psi).

### Immunofluorescence staining

Paraffin-embedded liver tissues were deparaffined and subjected to antigen retrieval using ethanol and citrate buffer (Servicebio, China). Samples were blocked with PBS containing 3% BSA for 30 min, followed by incubation overnight at 4 °C with TOMM20 and MtCo-1 primary antibodies, then with secondary antibodies at room temperature for 50 min. TSA staining (iF488-Tyramide, Servicebio, China) in dark at room temperature for 10 min, then cell nucleus were counterstained with DAPI for 10 min. Imaging was performed using a NIKON ECLIPSE C1 fluorescence microscope (NIKON, Japan).

### Statistical analysis

Statistical analyses were performed through GraphPad Prism software (V 9.5.1, La Jolla, CA, USA). All data were tested for normality and homogeneity of variance using Shapiro-Wilk test before one-way ANOVA and two-way ANOVA with Tukey’s multiple comparison test for post-hoc analysis. Data were presented as mean ± SEM and visualized using GraphPad Prism software. A *P*-value < 0.05 was considered statistically significant, and the sample size (*n*) for each group is indicated in the figure legends.

## Results

### DHA promotes LD clearance in primary bovine hepatocytes – in vitro

**Fig. 1:**
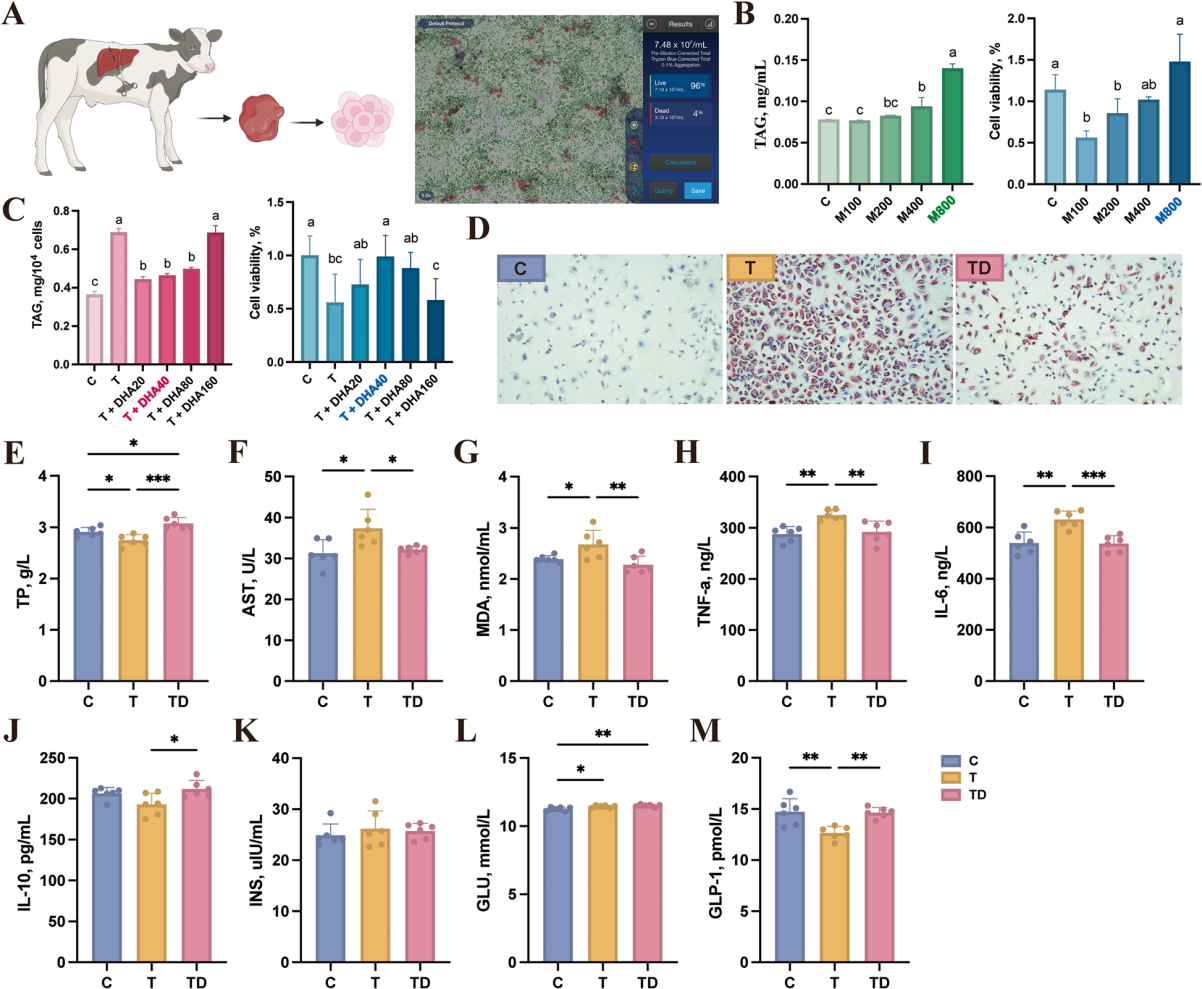
The supplementation of DHA on fatty liver cells alleviates lipid deposition. **A** Primary hepatic cell extraction and activity detection. **B** The change of TAG content (left) and cell viability (right) of primary hepatic cells after the treatment of different mixed acid (1 palmitic acid:2 oil acid) content (100, 200, 400, 800 µmol/L), and 800 µmol/L was selected for further analysis. **C** The change of TAG content (left) and cell viability (right) of primary hepatic cells after the treatment of different DHA content (20, 40, 80, 160 µmol/L), and 40 µmol/L was selected for further analysis. **D** Representative Oil Red O staining of primary hepatic cells. **E**–**M** Compare supernatant aspartate aminotransferase (AST), total protein (TP), malondialdehyde (MDA), tumor necrosis factor-α (TNF-α), interleukin-6 (IL-6), interleukin-10 (IL-10), glucagon-like peptide-1 (GLP-1), glucose (GLU) and insulin (INS) concentrations of primary hepatic cells after DHA intervention (*n* = 6). C group: healthy cells; T group: fatty liver cells; TD group: fatty liver cells supplemented DHA. ^a^^–^^c^Different letters indicate significant differences (*P* < 0.05). ^*^P < 0.05, ^**^P < 0.01, ^***^ *P* < 0.001

To accurately simulate the effects of DHA on hepatic lipid accumulation in dairy cows with fatty liver, we first isolated primary hepatocytes from calves using an optimized two-step collagenase perfusion protocol as described in method and materials (Fig. 1A). Subsequently, a fatty liver cell model was established by incubating hepatocytes with a mixture of 800 μmol/L PA and OA at a ratio of 1:2 for 12 h, as validated by assessments of cell viability and intracellular TAG accumulation (Fig. 1B). Upon treatment with graded concentrations of DHA, we observed that 20–80 μmol/L DHA for 12 h significantly reduced TAG content in steatotic hepatocytes, whereas 160 μmol/L DHA failed to elicit lipid-lowering effects (Fig. 1C). Considering cytotoxicity and efficacy, 40 μmol/L DHA was selected for subsequent in vitro investigations. Oil Red O staining corroborated this finding by visually demonstrating reduced LD accumulation in DHA-treated hepatocytes (Fig. 1D). Biochemical analysis revealed that steatotic hepatocytes exhibited significantly lower TP (Fig. 1F) content compared to healthy controls, which was notably restored following DHA supplementation. Besides, the concentrations of AST (Fig. 1E) and MDA (Fig. 1G) were markedly elevated in steatotic cells, but significantly attenuated by DHA treatment, indicating improved hepatic function and reduced oxidative stress. Inflammatory markers, including TNF-α (Fig. 1H) and IL-6 (Fig. 1I), were significantly increased in steatotic hepatocytes but suppressed following DHA exposure. Although IL-10 (Fig. 1J) content was statistically unchanged between healthy and steatotic groups, DHA intervention markedly increased it, suggesting enhanced anti-inflammatory capacity. Concurrently, DHA supplementation restored the markedly reduced GLP-1 (Fig. 1K) content, a key indicator of insulin resistance, in fatty liver cells, indicating that DHA may ameliorate lipid-induced insulin resistance.

### DHA modulates lipid metabolism genes and mitochondrial function – in vitro

**Fig. 2:**
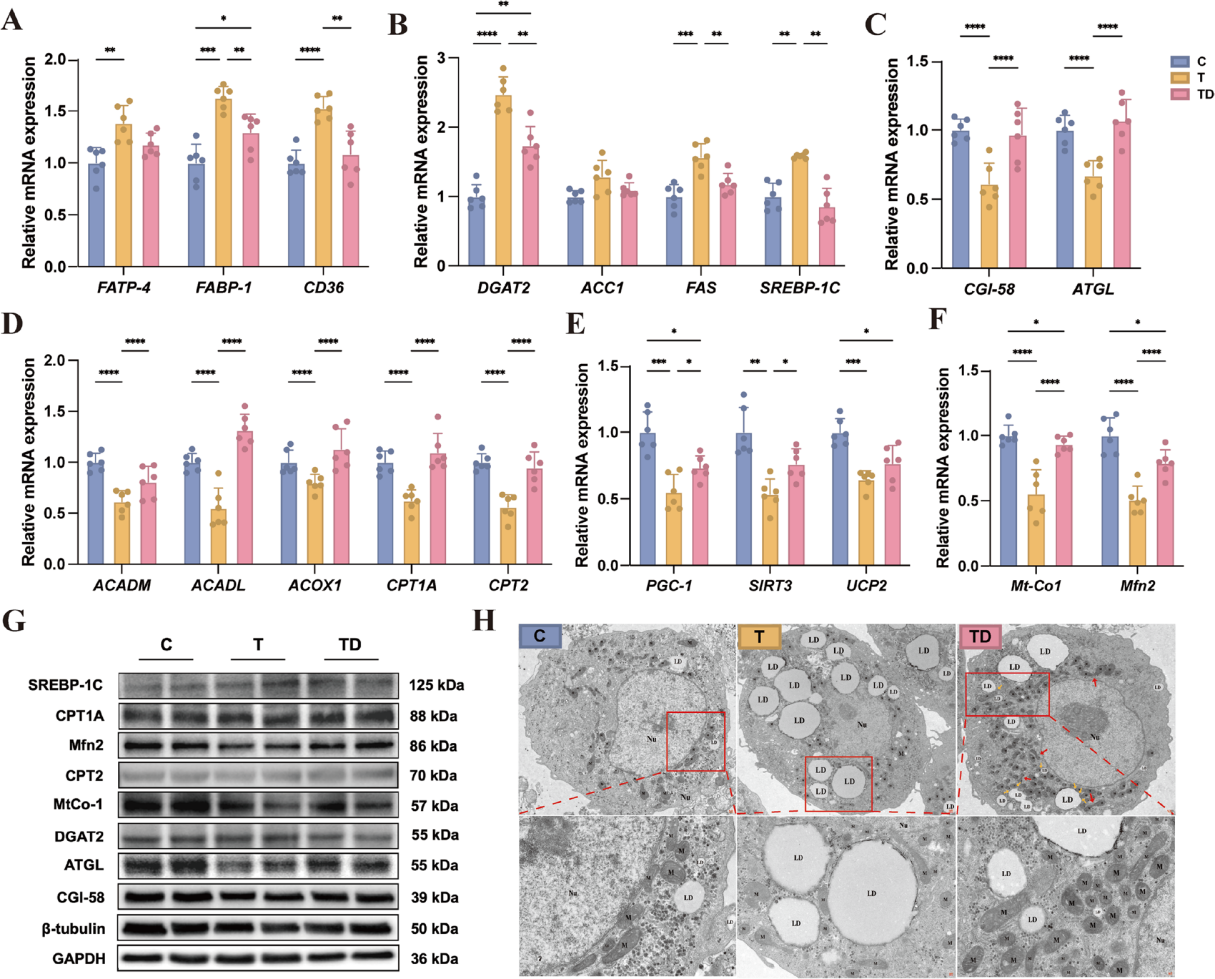
Lipid metabolic gene and mitochondrial status change in fatty liver cells after the supplementation of DHA. **A**–**F** Compare changes of gene expression involved in fatty acid transport (**A**), lipogenesis (**B**), lipolysis (**C**), fatty acid oxidation (**D**), energy metabolism (**E**) and mitochondrial function (**F**) from primary hepatic cells after DHA intervention (*n* = 6). **G** Changes of protein expression in primary hepatic cells after DHA treatment. **H** Transmission electron microscopy (TEM) images the mitochondria and lipid droplet in primary hepatic cells after DHA supplementation. C group: healthy cells; T group: fatty liver cells; TD group: fatty liver cells supplemented DHA. The red box and dotted lines in panel H shows representative lipid droplets accumulation and mitochondrial morphology in each group. ^*^P < 0.05, ^**^P < 0.01, ^***^P < 0.001 and ^****^P < 0.0001

Transcriptomic profiling revealed that steatotic hepatocytes exhibited significant upregulation of genes governing fatty acid transport (Fig. 2A; *FABP-1*, *CD36*) and lipid synthesis (Fig. 2B; *DGAT2*, *FAS*, *SREBP-1C*). Conversely, genes associated with lipolysis (Fig. 2C; *CGI-58*, *ATGL*), fatty acid oxidation (Fig. 2D; *ACADM*, *ACADL*, *ACOX1*, *CPT1A*, *CPT2*), energy metabolism (Fig. 2E; *PGC-1*, *SIRT3*) and mitochondrial function (Fig. 2F; *Mt-Co1*, *Mfn2*) were significantly downregulated compared to healthy controls. DHA supplementation effectively reversed these transcriptional alterations, indicating its broad regulatory effects on hepatic lipid metabolism pathways. Protein-level analyses by western blot supported the gene expression results (Fig. 2G). Of note, although *Mt-Co1* and *Mfn2* genes were partially restored, they remained significantly lower than those in healthy hepatocytes, suggesting incomplete but substantial recovery of mitochondrial function. Given the critical role of mitochondria in lipid catabolism and energy metabolism, we further assessed mitochondrial ultrastructure using TEM. As shown in Fig. 2H, steatotic hepatocytes displayed severe mitochondrial abnormalities, including cristae disorganization and nuclear membrane blurring, accompanied by excessive and enlarged LDs. In contrast, DHA treatment restored mitochondrial structural integrity, promoted organellar fusion, and enhanced LD-mitochondria contact events. These findings suggest that DHA not only modulates gene and protein expression related to lipid metabolism, but also improves mitochondrial architecture and function, facilitating more efficient lipid utilization in hepatocytes subjected to steatotic stress.

### DHA mediates LD clearance in liver of dairy cows – in vivo

**Fig. 3:**
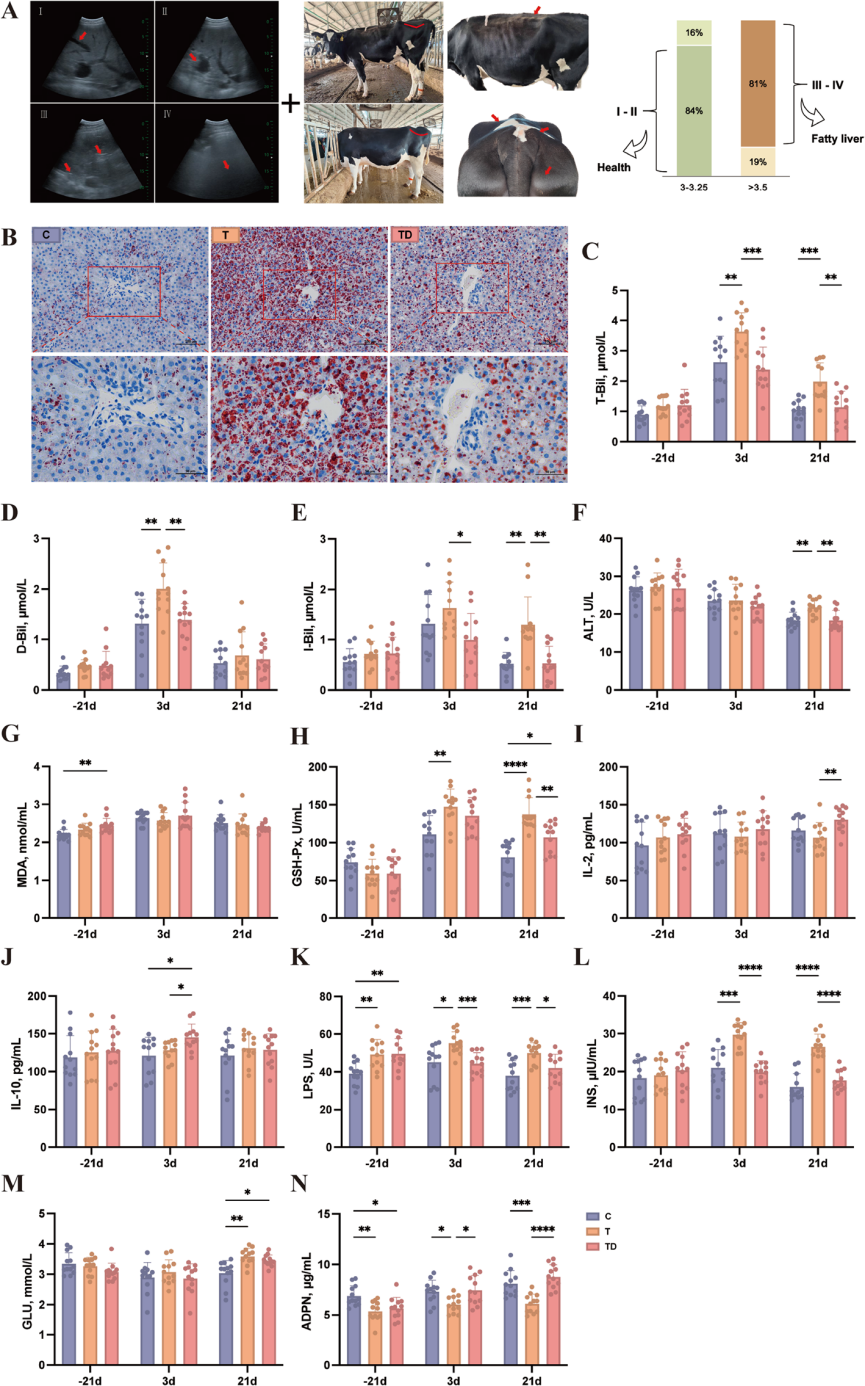
Dietary DHA supplementation alleviates hepatic lipid deposition and improves blood characteristics. **A** Dairy cows grouping criteria. **B** Representative Oil Red O staining of dairy cows’ liver. **C**–**N** Compare blood total bilirubin (T-Bil), direct bilirubin (D-Bil), indirect bilirubin (I-Bil), alanine aminotransferase (ALT), malondialdehyde (MDA), glutathione peroxidase (GSH-Px), interleukin-2 (IL-2), interleukin-10 (IL-10), lipopolysaccharide (LPS), insulin (INS), glucose (GLU), adiponectin (ADPN) concentrations of dairy cows after dietary DHA supplementation (*n* = 12). The guideline of hepatic health grade based on ultrasound imaging: Grade I (predicted health): homogeneous hepatic echotexture with clearly defined vascular contours and full penetration of ultrasound beams to the deep parenchyma; Grade II (predicted mild fat deposition): blurred vascular margins with relatively uniform parenchymal echoes and preserved beam penetration; Grade III (predicted moderate fat deposition): loss of vascular visibility, marked inhomogeneous echogenicity and scattered hyperechoic foci appearing before reaching the deep parenchyma; Grade IV (predicted severe fat deposition): complete loss of vascular visibility and failure of ultrasound beam penetration, indicating diffuse fat infiltration with pronounced acoustic attenuation. The ratio of 84% animals simultaneously met the criteria of ultrasound score of I-II and BCS at 3.0–3.25, which were predicted high possible to keep health after calving, whereas ratio of 81% animals simultaneously met the criteria of ultrasound score of III-IV and BCS higher than 3.5, which were predicted to be at high risk of developing fatty liver disease after calving. The red box and dotted lines in panel B shows representative lipid droplets accumulation in each group. C group: healthy cows; T group: fatty liver cows; TD group: fatty liver cows supplemented DHA. ^*^P < 0.05, ^**^P < 0.01, ^***^P < 0.001 and ^****^P < 0.0001

To evaluate the hepatoprotective effects of DHA in vivo, dairy cows were classified into three experimental groups: healthy control (C), fatty liver (T) and fatty liver supplemented with DHA (TD) based on BCS and hepatic ultrasonography detection (Fig. 3A). Due to ethical constraints, liver biopsies were performed once between 7 and 14 days after calving. Histological evaluation using Oil Red O staining demonstrated a marked increase in LD accumulation in the livers of fatty liver cows compared to healthy controls. Dietary supplementation with rumen-protected DHA markedly reduced lipid deposition (Fig. 3B), suggesting a clear ameliorative effect of DHA on hepatic lipid overload. Biochemical indices of liver function further supported these observations. Serum T-bil (Fig. 3C) and I-bil (Fig. 3E) concentrations were significantly lower in the TD group than in the T group on day 21 postpartum. The concentration of ALT (Fig. 3F) showed no significant differences among groups at day 21 before calving, but both the C and TD groups exhibited significantly lower ALT contents than the T group at day 21 postpartum. Markers of oxidative stress demonstrated that the difference of MDA concentration before calving between C and TD group disappeared after DHA supplementation (Fig. 3G). While GSH-Px (Fig. 3H) content showed no significant difference at baseline, its level was markedly reduced in the TD group relative to the T group on day 21 postpartum. Regarding inflammatory status, animals supplemented with DHA exhibited significantly elevated IL-10 content on day 3 postpartum (Fig. 3J). Concurrently, LPS (Fig. 3K) content was initially elevated in T and TD groups compared to control, but significantly suppressed after DHA supplementation on both day 3 and 21 postpartum. Additionally, DHA intervention significantly reduced insulin content (Fig. 3L) on days 3 and 21 postpartum, despite no significant changes in glucose concentration (Fig. 3M). Notably, ADPN, a key biomarker of insulin sensitivity, was markedly increased in the TD group at both postpartum time points, although prepartum ADPN content was higher in healthy cows than in both diseased groups (Fig. 3N). Collectively, these findings demonstrate that dietary DHA supplementation not only mitigates hepatic lipid accumulation in postpartum dairy cows but also improves liver function, reduces oxidative stress and inflammation, and enhances insulin sensitivity.

### DHA regulates PUFAs profiles in milk and plasma – in vivo

**Fig. 4:**
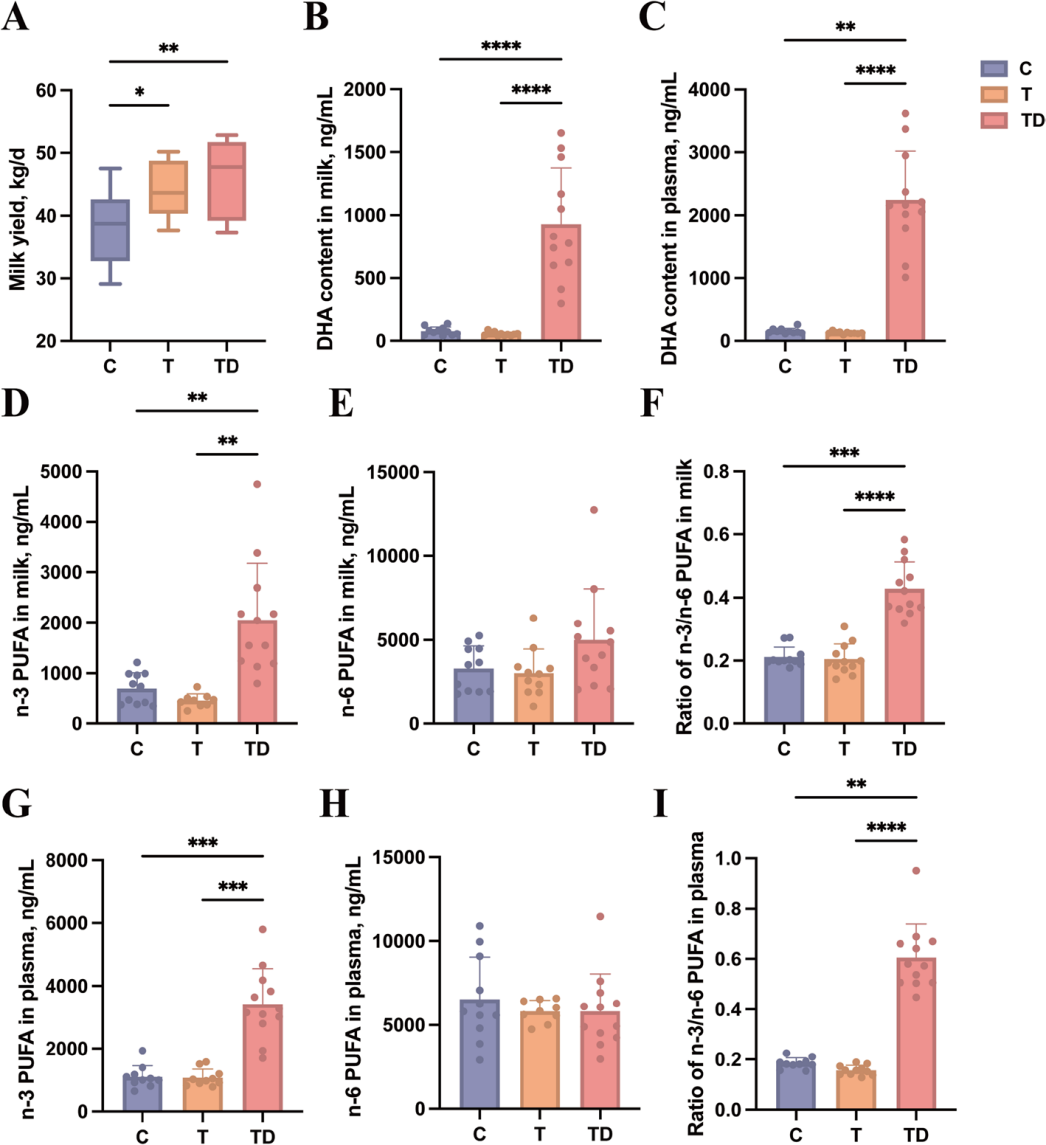
Dietary DHA supplementation changes n3/n6 ratio in milk and plasma at 21 days postpartum. **A** Total milk yield after calving until 21 days. **B** The DHA content in milk. **C** The DHA content in plasma. **D** The n-3 PUFA content in milk. **E** The n-6 PUFA content in milk. **F** Ratio of n-3/n-6 PUFA in milk. **G** The n-3 PUFA content in milk plasma. **H** The n-6 PUFA content in plasma. **I** Ratio of n-3/n-6 PUFA in plasma. *n* = 12. C group: healthy cows; T group: fatty liver cows; TD group: fatty liver cows supplemented DHA. n-3 includes alpha-Linolenic acid (C18:3, ALA), Eicosapentaenoic acid (C20:5, EPA), Docosapentaenoic acid (C22:5, DPA), Docosahexaenoic acid (C22:6, DHA); n-6 includes Linoleic acid (C18:2, LA), gamma-Linolenic acid (C18:3, GLA), homo-gamma-Linolenic acid (C20:3, DGLA), Arachidonic acid (C20:4, AA), Linoelaidic acid (C18:2, CLA). ^*^P < 0.05, ^**^P < 0.01, ^***^P < 0.001 and ^****^P < 0.0001

Postpartum milk yield was monitored daily. Compared to healthy animal, dairy cows with fatty liver produced more milk (Fig. 4A), suggesting intensified metabolic stress and exacerbated NEB, potentially driving ectopic lipid accumulation and inflammatory cascades. Notably, the DHA supplemented group maintained high milk yield while exhibiting reduced hepatic lipid accumulation, indicating improved metabolic adaptation. To verify the bioavailability of dietary DHA, we quantified DHA content in milk (Fig. 4B) and plasma (Fig. 4C); results revealed a large increase of DHA content in both samples after rumen-protected algal power supplementation, confirming effective systemic absorption. Furthermore, n3 PUFAs (Fig. 4D) and n3/n6 PUFAs ratio (Fig. 4F) in milk demonstrated markedly significant elevations in TD group, while n6 PUFAs (Fig. 4E) suggested no significant difference among three groups. Similar patterns were observed in plasma (Fig. 4G–I). A higher n-3/n-6 PUFA ratio is associated with reduced inflammation and enhanced metabolic health in both animals and humans [26]. Thus, the altered PUFA profiles in response to DHA likely contributed to the observed improvements in hepatic lipid metabolism and inflammatory status. Additionally, the enrichment of milk with DHA and n-3 PUFAs may offer additional value for the exploration of functional dairy products.

### DHA modulates hepatic lipid metabolism – in vivo

**Fig. 5:**
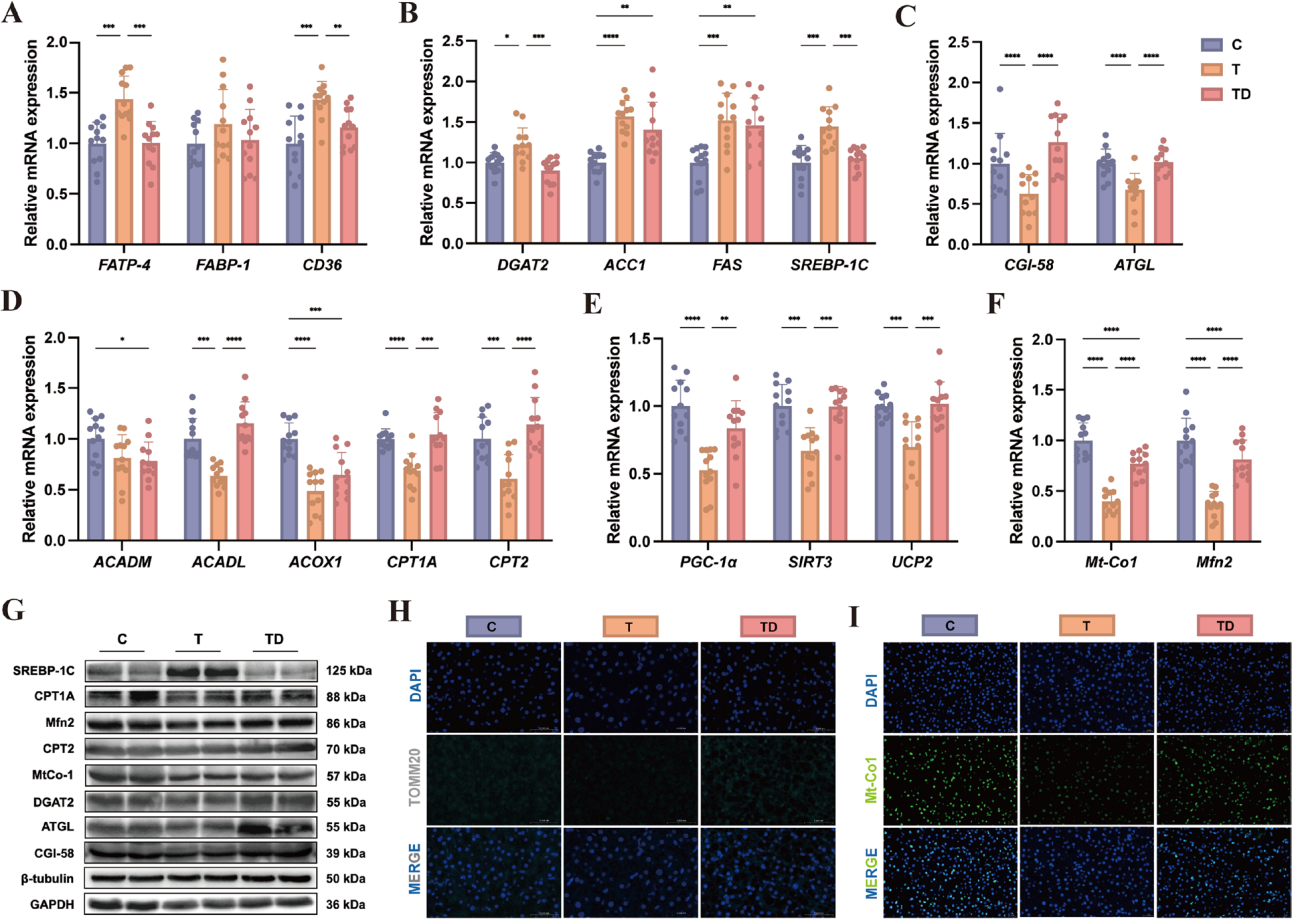
Dietary DHA supplementation modulates hepatic lipid metabolism. **A**–**F** Compare changes of gene expression involved in fatty acid transport (**A**), lipogenesis (**B**), lipolysis (**C**), fatty acid oxidation (**D**), energy metabolism (**E**) and mitochondrial function (**F**) from dairy cows’ liver after dietary DHA supplementation (*n* = 12). **G** Changes of protein expression in dairy cows’ liver after dietary DHA supplementation. **H** Immunofluorescence analysis of TOMM20 expression in live tissues after dietary DHA supplementation. **I** Immunofluorescence analysis of Mt-Co1 expression in live tissues after dietary DHA supplementation. C group: healthy cows; T group: fatty liver cows; TD group: fatty liver cows supplemented DHA. ^*^P < 0.05, ^**^P < 0.01, ^***^P < 0.001 and ^****^P < 0.0001

Consistent with in vitro findings, dietary DHA supplementation significantly modulated hepatic lipid metabolism in dairy cows. qPCR analysis revealed that DHA downregulated the expression of genes associated with fatty acid transport, including *FATP-4* and *CD36* (Fig. 5A), and lipid synthesis, such as *DGAT2* and *SREBP-1C* (Fig. 5B). Conversely, genes involved in lipolysis (*CGI-58*, *ATGL*; Fig. 5C), fatty acid oxidation (*ACADL*, *CPT1A*, *CPT2*; Fig. 5D), energy metabolism (*PGC-1*, *SIRT3*, *UCP2*, Fig. 5E) and mitochondrial function (*Mt-Co1*, *Mfn2*; Fig. 5F) were upregulated in the TD group compared to the untreated fatty liver group. Western blot analysis further confirmed these gene expression changes at the protein level (Fig. 5G). Additionally, to examine subcellular localization of key mitochondrial proteins, we employed immunofluorescence staining to analyze TOMM20 (Fig. 5H) and Mt-Co1 (Fig. 5I). Results showed that the fluorescence intensity for TOMM20 and Mt-Co1 were significantly attenuated in the T group, indicating diminished mitochondrial quantity and function. Conversely, both markers were markedly enhanced in the TD group, albeit not fully restored to normal levels. These findings further corroborate the aforementioned observations from qPCR and WB analyses, suggesting DHA’s partial mitigation of mitochondrial dysfunction caused by fatty liver.

## Discussion

Energy stress in transition dairy cows contributes to hepatic lipidosis [27]. While conventional strategies are more focused on resolving downstream problems such as improving lactation performance and antioxidant capacity, along with attenuating inflammatory responses [28–30], neglect early nutritional intervention targeting hepatic lipid clearance. In this study, our complementary in vitro and in vivo models consistently demonstrate that DHA supplementation can effectively reconstitute mitochondrial function and regulate key lipid metabolism gene networks, thereby alleviating hepatic lipid deposition and resolving metabolic disturbances including inflammation, oxidative stress, and insulin resistance. In our knowledge, this study is the first to demonstrate the hepatoprotective effects of DHA in transition dairy cows.

Mitochondria are considered as the energy factories of cells, where many vital metabolic reactions occur [31, 32]. As a metabolic hub, the liver is highly dependent on mitochondria to meet its energy needs in the form of ATP, which in turn influences and regulates energy metabolism [33]. Indeed, there is accumulating evidence that mitochondria are central organelles in the pathogenesis of metabolic dysfunction-associated liver disease [34, 35]. In our trial, TEM results revealed that DHA intervention reversed pathological mitochondrial alterations in steatotic hepatocytes, as evidenced by cristae reconstruction, improved mitochondrial morphology and number, frequent fusion and contact with LD events. More specifically, mitochondria connected with LDs have unique bioenergetics characteristics [36]. Previous study suggested that glycolytic enzyme PFKL governed lipolysis by promoting LD-mitochondria tethering to enhance β-oxidation and tumor cell proliferation [37]. Miner et al. reported that PLIN5 interacts with FATP-4 at membrane contact sites to promote LD to mitochondria fatty acid transport [38]. Hu et al. also believed that Mfn2/Hsc70 complex mediates the formation of mitochondria-LD membrane contact and regulates myocardial lipid metabolism [39]. Indeed, the increased LD-mitochondria coupling after DHA intervention plays a critical role in promoting fatty acid oxidation, which is further supported by our gene and protein expression results. It may be one of the most direct and rapid mechanisms for accelerating lipid utilization and reducing lipid deposition in current study.

Furthermore, mitochondria are highly adaptable organelles, as two mitochondria can form a single, tubular like mitochondrion by fusing their outer and inner membranes to maintain the integrity of mitochondrial function and enhance cellular energy supply [40]. Upon DHA supplementation to hepatocytes, significant upregulation of Mfn2 expression was observed at both transcriptional and translational levels, suggesting that DHA may promote mitochondrial fusion, which may be related to enhanced Mfn2 expression. Mitochondrial fusion establishes stronger oxidative phosphorylation capacity and a healthier mitochondrial network, thereby augmenting cellular metabolic efficiency [41]. Additionally, TOMM20, an outer mitochondrial membrane protein commonly used as a marker for mitochondrial morphology, distribution, and quantity [42]. Our immunofluorescence staining images of TOMM20 from dairy cows’ liver suggested that the quantity of mitochondria reduced after fatty liver incidence, but DHA promoted mitochondrial biogenesis. Although previous work suggested that greater numbers of mitochondria was observed during fatty liver, it may be a compensatory manifestation [23]. In cases of severe fatty liver, the opposite result will be found. MtCo-1, also known as COX-1, is the final enzyme of the electron transport chain of mitochondrial oxidative phosphorylation and serve as a biomarker for both the presence of mitochondria and functional mitochondrial respiration [43]. The result of MtCo-1 in our study suggested that DHA may be associated with promoting mitochondrial biogenesis and repaired mitochondrial dysfunction induced by lipid deposition, thereby enhancing β-oxidation of fatty acids. Moreover, DHA supplementation also downregulated genes associated with lipid synthesis, thereby reducing triglyceride accumulation. Concurrently, DHA upregulated genes involved in lipid breakdown to promote lipolysis. These effects, along with enhanced fatty acid oxidation, collectively mitigated lipid deposition. Future studies will focus on a deeper mechanistic understanding, particularly the roles of Mfn2 and TOMM20 in the regulation of mitochondrial dynamics and lipid metabolism using siRNA technique, to better understand how DHA alleviates fatty liver incidence.

Prior studies suggested that the incidence of fatty liver is frequently associated with pathological processes such as inflammation, oxidative stress, and insulin resistance [44–46], findings from both in vivo and in vitro results in current study further supported above conclusion. DHA supplementation attenuated lipid deposition-induced inflammation and oxidative stress through restoring mitochondrial integrity and modulating mitochondrial biogenesis and function. However, a limitation of this study is the absence of a healthy + DHA group, which prevents a full assessment of DHA’s metabolic effects in non-diseased cows. Future studies using a 2 × 2 factorial design (health × DHA) are warranted to further clarify the preventive role of DHA. Additionally, the observed mitigation of inflammation and oxidative stress may also be attributed to the compositional shifts in plasma PUFA composition, as reflected by elevated n-3 PUFA and n-3/n-6 ratio [47–49]. Notably, in transition dairy cows susceptible to fatty liver disease, the benefits of DHA intervention extend beyond animal health. It also enriched milk with n-3 PUFAs and n-3/n-6 ratio, supplying additional value for human nutrition [13]. This finding is consistent with recommendations from the World Health Organization and the Food and Agriculture Organization (FAO/WHO), which advocate for increased dietary n-3 PUFA intake to promote metabolic and cardiovascular health.

## Conclusion

In conclusion, our study demonstrates that DHA effectively alleviates hepatic lipid deposition and associated inflammation, oxidative stress, and insulin resistance by remodeling mitochondrial dynamics and reprogramming lipid metabolism gene expression. Additionally, DHA modulates systemic and milk PUFA profiles by increasing n-3 PUFAs. A key highlight of this study lies in the integration of in vitro and in vivo models, allowing for mechanistic validation and physiological confirmation. These findings support the use of rumen-protected DHA as a promising nutritional strategy for the prevention of spontaneous fatty liver in dairy cows. Beyond animal health, DHA supplementation also enhances the nutritional quality of milk, offering potential benefits for the development of functional dairy products and human health.

## Supplementary Information

Additional file 1: Table S1. Commercial assay information. Table S2. The PCR primers design in this study. Table S3. The detailed antibodies information.

## Data Availability

The datasets used and analyzed during the current study are available from the corresponding author on reasonable request.

## Abbreviations

ACADL: Acyl-CoA dehydrogenase long chain
ACADM: Acyl-CoA dehydrogenase medium chain
ACC1: Acetyl-CoA carboxylase 1
ACOX1: Acyl-CoA oxidase 1
ADPN: Adiponectin
ALT: Alanine aminotransferase
AST: Aspartate aminotransferase
ATGL: Patatin like phospholipase domain containing 2
BCS: Body condition score
CD36: CD36 molecule
CGI-58: Abhydrolase domain containing 5
CPT1A: Carnitine palmitoyltransferase 1A
CPT2: Carnitine palmitoyltransferase 2
D-Bil: Direct bilirubin
DGAT2: Diacylglycerol O-acyltransferase 2
DHA: Docosahexaenoic acid
DMI: Dry matter intake
FABP-1: Fatty acid binding protein 1
FAS: Fas cell surface death receptor
FATP-4: Solute carrier family 27 member 4
GLP-1: Glucagon-like peptide-1
GLU: Glucose
GSH-Px: Glutathione peroxidase
I-Bil: Indirect bilirubin
IL-2: Interleukin-2
IL-6: Interleukin-6
IL-10: Interleukin-10
INS: Insulin
LD: Lipid droplet
LPS: Lipopolysaccharide
MAFLD: Metabolic-associated fatty liver disease
MDA: Malondialdehyde
Mfn2: Mitofusin 2
Mt-Co1: Mitochondrially encoded cytochrome c oxidase I
NEB: Negative energy balance
OA: Oleic acid
PA: Palmitic acid
PGC-1: Phosphatidylglycerol phospholipase
PUFA: Polyunsaturated fatty acid
SIRT3: Sirtuin 3
SREBP-1C: Sterol regulatory element binding transcription factor 1
TAG: Triglyceride
T-Bil: Total bilirubin
TEM: Transmission electron microscopy
TMR: Total mixed ration
TNF-α: Tumor necrosis factor-α
TP: Total protein
UCP2: Uncoupling protein 2
