# Supplementary material for: Docosahexaenoic acid (DHA) alleviates hepatic lipid deposition in dairy cows during the transition period: an integrated in vitro and in vivo study
Source: J Anim Sci Biotechnol. 2025 Dec 5;16:166. doi: 10.1186/s40104-025-01308-4 (PMC12679754; doi:10.1186/s40104-025-01308-4)
Supplement: Supplementary file 1 — Additional file 1: Table S1. Commercial assay information. Table S2. The PCR primers design in this study. Table S3. The detailed antibodies information. [file 40104_2025_1308_MOESM1_ESM.docx]

**Table S1** Commercial assay information

| **Item** | **Source** | **Identifier** |
| --- | --- | --- |
| ALT Detection Kit | Shenzhen Mindray Medical Equipment Co., Ltd. | 105-000442-00 |
| AST Detection Kit | Shenzhen Mindray Medical Equipment Co., Ltd. | 105-000445-00 |
| GLU Detection Kit | Shenzhen Mindray Medical Equipment Co., Ltd. | 105-000450-00 |
| Tbil Detection Kit | Shenzhen Mindray Medical Equipment Co., Ltd. | 105-000454-00 |
| Dbil Detection Kit | Shenzhen Mindray Medical Equipment Co., Ltd. | 105-000455-00 |
| TP Detection Kit | Shenzhen Mindray Medical Equipment Co., Ltd. | 105-015578-00 |
| Bovine TNF-a Elisa Kit | Beijing Laibotairui Technology Co. Ltd. | CK-E92026 |
| Bovine IL-2 Elisa Kit | Beijing Laibotairui Technology Co., Ltd. | CK-E92642 |
| Bovine IL-6 Elisa Kit | Beijing Laibotairui Technology Co., Ltd. | CK-E92031 |
| Bovine IL-10 Elisa Kit | Beijing Laibotairui Technology Co., Ltd. | CK-E92644 |
| Bovine GLP-1 Elisa Kit | Beijing Laibotairui Technology Co., Ltd. | CK-E99910 |
| Bovine insulin Elisa Kit | Beijing Laibotairui Technology Co., Ltd. | CK-E92021 |
| Bovine LPS Elisa Kit | Beijing Laibotairui Technology Co., Ltd. | CK-E90093 |
| Bovine Adiponectin Elisa Kit | Beijing Laibotairui Technology Co., Ltd. | CK-E95611 |
| Glutathione Peroxidase (GSH-P_X_) assay kit (Colorimetric method) | Nanjing Jiancheng Bioengineering Institute | A005-1-2 |
| Malondialdehyde (MDA) assay kit (TBA method) | Nanjing Jiancheng Bioengineering Institute | A003-1-2 |

**Table S2** The PCR primers design in this study

| **Gene** | **Forward (5´→3´)** | **Reverse (5´→3´)** |
| --- | --- | --- |
| *FATP-4* | CAGCCGTCCCAACAAGG | TGATACAGTCGTCCCAGAAGC |
| *FABP-1* | GAGCCTGGTCAAGTTCTGTTCTGG | GATGTTCCCTGCCGAGTGGTA |
| *CD36* | GGATTTACTTTACGGTTTG | ACATTTCCGCCTTCTC |
| *DGAT2* | CCAAGTCATCTCGGTGCTACA | TGACCTCCTGCCACCTTTC |
| *ACC1* | GGAGGAGGGAAGGGAATCAG | GCACAGAGCAATCACGACCA |
| *FAS* | CCTCCTCATCCCAATAGTTC | TTAAATTCAGTTGCCTCCCT |
| *SREBP-1C* | TTGAATAAATCTGCCGTCTTGC | CCACTTCCACCGCTGCTACT |
| *CGI-58* | CGACCCAGATTTGACAGT | TAACCAGCAGCCAGGAAC |
| *ATGL* | GGTGCCCTACACTCTGC | CTGCCTGTCTGCTCCTT |
| *ACADM* | CTGAAATGGCAATGAAAG | GCAATAGAGGCATAATAGGT |
| *ACADL* | AGATAGCAGTTTCAGCCAGTG | CAGATGCCCAGTATTTCG |
| *ACOX1* | AGCCTTTGCCAGGTATTA | TCCCGTAGGTCAGCTTGTTA |
| *CPT1A* | CCTTCCCATTCCGCACTTTC | CGGTCTCCGTTCTGCCCTCT |
| *CPT2* | CGACACTTGTTTGCTCTGCGGTAC | GGGCATTGCGGCTTTGGT |
| *Mt-Co1* | CCATGCAGGAGCTTCAGT | GCTAATACAGGGAGCGAGA |
| *Mfn2* | TGGTCCTCAAGGTTTACAAGA | TTCAAGCCGTCAATCATCT |
| *PGC-1α* | ACCAGTGGACACGAGGAAAG | CAGGTAGCACGGGTCGGAAT |
| *SIRT3* | ATGGCGTTGTTTCCTCGTTC | ACTTCTTCTGACTGTGGTCTCCC |
| *UCP2* | GGGGACTCTGGAAAGGGACA | AGGCAGAAGTAAAGTGGCAAGG |

**Table S3** The detailed antibodies information

| **Antibodies** | **Source** | **Identifier** |
| --- | --- | --- |
| Rabbit anti-SREBP-1c Polyclonal Antibody | Proteintech | Cat# 14088-1-AP |
| Rabbit anti- CPT-1A Polyclonal antibody | Proteintech | Cat# 15184-1-AP |
| Rabbit anti-Mfn2 Polyclonal antibody | Proteintech | Cat# 12186-1-AP |
| Rabbit anti-CPT2 Polyclonal antibody | Proteintech | Cat# 26555-1-AP |
| Rabbit anti-Mt-Co1 Polyclonal antibody | Bioss | Cat# bs-3953R |
| Rabbit anti-DGAT2 Polyclonal Antibody | Absin | Cat# abs146950 |
| Goat anti-ATGL Polyclonal antibody | RayBiotech | Cat# ER-14-0199 |
| Rabbit anti-CGI-58 Polyclonal Antibody | Bioss | Cat# bs-5028R |
| ‌Rabbit anti-**β-t**ubulin Monoclonal Antibody | Bioss | Cat# bsm-33034R |
| Mouse anti-GAPDH Monoclonal Antibody | Proteintech | Cat# 60004-1-Ig |
| Goat anti-rabbit, HRP-labeled | Beyotime | Cat# A0208 |
| Goat anti-mouse, HRP-labeled | Beyotime | Cat# A0216 |
| Rabbit anti-goat, HRP-labeled | Proteintech | Cat# SA00001-4 |
